# Identification and Validation of a Novel Locus Controlling Spikelet Number in Bread Wheat (*Triticum aestivum* L.)

**DOI:** 10.3389/fpls.2021.611106

**Published:** 2021-02-26

**Authors:** Tao Li, Guangbing Deng, Yanyan Tang, Yan Su, Jinhui Wang, Jie Cheng, Zhao Yang, Xuebing Qiu, Xi Pu, Haili Zhang, Junjun Liang, Maoqun Yu, Yuming Wei, Hai Long

**Affiliations:** ^1^Triticeae Research Institute, Sichuan Agricultural University, Chengdu, China; ^2^Chengdu Institute of Biology, Chinese Academy of Sciences, Chengdu, China; ^3^State Key Laboratory of Crop Gene Exploration and Utilization in Southwest China, Chengdu, China

**Keywords:** wheat (*Triticum aestivum* L.), QTL, linkage analysis, spikelet number, KASP, yield

## Abstract

Spikelet number is an important target trait for wheat yield improvement. Thus, the identification and verification of novel quantitative trait locus (QTL)/genes controlling spikelet number are essential for dissecting the underlying molecular mechanisms and hence for improving grain yield. In the present study, we constructed a high-density genetic map for the Kechengmai1/Chuanmai42 doubled haploid (DH) population using 13,068 single-nucleotide polymorphism (SNP) markers from the Wheat 55K SNP array. A comparison between the genetic and physical maps indicated high consistence of the marker orders. Based on this genetic map, a total of 27 QTLs associated with total spikelet number per spike (TSN) and fertile spikelet number per spike (FSN) were detected on chromosomes 1B, 1D, 2B, 2D, 3D, 4A, 4D, 5A, 5B, 5D, 6A, 6B, and 7D in five environments. Among them, five QTLs on chromosome 2D, 3D, 5A, and 7D were detected in multiple environments and combined QTL analysis, explaining the phenotypic variance ranging from 3.64% to 23.28%. Particularly, *QTsn/Fsn.cib-3D* for TSN and FSN [phenotypic variation explained (PVE) = 5.97–23.28%, limit of detection (LOD) = 3.73–18.51] is probably a novel locus and located in a 4.5-cM interval on chromosome arm 3DL flanking by the markers *AX-110914105* and *AX-109429351.* This QTL was further validated in other two populations with different genetic backgrounds using the closely linked Kompetitive Allele-Specific PCR (KASP) marker *KASP_AX-110914105*. The results indicated that *QTsn/Fsn.cib-3D* significantly increased the TSN (5.56–7.96%) and FSN (5.13–9.35%), which were significantly correlated with grain number per spike (GNS). We also preliminary analyzed the candidate genes within this locus by sequence similarity, spatial expression patterns, and collinearity analysis. These results provide solid foundation for future fine mapping and cloning of *QTsn/Fsn.cib-3D*. The developed and validated KASP markers could be utilized in molecular breeding aiming to increase the grain yield in wheat.

## Introduction

Bread wheat (*Triticum aestivum* L.) is one of the most important stable foods in the world, providing more than 20% of the calories and protein in our daily diet (Yao et al., 2019; Liu et al., 2020). It was reported that world population is expected to reach nine billion by 2050, which will require raising overall food production by at least 70% to fulfill future food demand (Yang et al., 2012). Conversely, arable land and water resources for agriculture continue to dwindle, and climate change also causes adverse effects on crop productivity (Evans et al., 2008; Butterworth et al., 2010; Ceccarelli et al., 2010). Thus, increasing wheat yield is an urgent task to fulfill global food and nutritional security.

Grain yield of wheat is a complex trait affected by a multitude of genetic and environmental factors and is usually constituted of three major components, i.e., thousand kernel weight (TKW), grain number per spike (GNS), and spike number per unit area (Wang et al., 2018; Kuzay et al., 2019). GNS potential is largely established by the spike architecture. Similar to other Triticeae species, wheat contains an unbranched inflorescence, and a number of spikelets are directly attached to the inflorescence axis in a distichous phyllotaxis, with a terminal spikelet at its apex (Koppolu and Schnurbusch, 2019). The spikelet is made of an indeterminate number of florets attached to a secondary axis (rachilla) (Hanif and Langer, 1972; Wolde et al., 2019). Therefore, the number of fertile spikelet number per spike (FSN) and fertile floret per spikelet ultimately determined GNS (Guo et al., 2017; Golan et al., 2019). Previous studies have showed that floret fertility is severely affected by environmental and genetic factors as an abortion process of floral structures existing continuously during the whole floral developmental process (Guo and Schnurbusch, 2015; Sakuma et al., 2019). By comparison, spikelet number is determined at an early stage of wheat reproductive development, exhibiting less environmentally sensitive with high heritabilities (Zhang et al., 2018; Gao et al., 2019). Thus, understanding the genetic factors underlying spikelet number would provide the prerequisite information necessary to improve wheat yield.

Similar to other yield-related traits, spikelet number is a complicated quantitative trait [quantitative trait locus (QTL)] controlled by multiple genes in wheat. Thus, determination of the number, chromosomal localization, and genetic effects of these polygenes is desirable for obtaining optimal genotype in breeding practice (Ma et al., 2007; Chen et al., 2020). However, due to the complexity of wheat genome, only a few genes associated with spikelet number or spike morphology have been cloned through homologous cloning approach. For instance, *WAPO1*, an ortholog of the rice gene *APO1*, on chromosome 7AL could regulate spikelet number by effect inflorescence development (Kuzay et al., 2019; Voss-fels et al., 2019). The domestication gene *Q* on chromosome 5A encodes a member of *AP2* transcription factor family and can regulate rachis fragility, glume tenacity, head length, and spikelet density (Faris et al., 2003; Simons et al., 2006; Debernardi et al., 2017; Greenwood et al., 2017; Jiang et al., 2019). The photoperiod sensitivity gene *Ppd-1* on chromosome 2D is associated with multiple pollinator traits, including spikelet number, spike length (SL), and number of days to heading (Okada et al., 2019); *TaMOC1*, an ortholog gene of rice *MOC1*, is significantly associated with spikelet number (Zhang et al., 2015); *FZ*P gene can drive supernumerary spikelet (Dobrovolskaya et al., 2015); *TaDEP1*, an ortholog of rice *DEP1*, relates to increase SL and reduces the spikelet number (Huang et al., 2009).

In addition, numerous QTLs for spikelet number have been identified on almost all the 21 chromosomes in wheat using different genetic populations and natural varieties (Cui et al., 2012; Yu et al., 2014; Gao et al., 2015; Luo et al., 2016; Zhai et al., 2016; Deng et al., 2017; Liu et al., 2018b; Wang et al., 2018; Koppolu and Schnurbusch, 2019; Ma et al., 2019; Yao et al., 2019; Kuang et al., 2020). Nevertheless, only a few of QTLs have been genetically verified, offering a foundation for fine mapping and map-based cloning, which greatly restrict the dissection of the molecular mechanisms underlying spikelet number as well as improvement of spikelet number in wheat breeding. Thus, the identification and verification of novel QTL/genes for spikelet number are vital.

In this study, we constructed a high-density genetic map using the Wheat 55K single-nucleotide polymorphism (SNP) array and a doubled haploid (DH) population derived from a cross between two wheat elite varieties K1 and CM42. QTL mapping for two spikelet number-related traits, total spikelet number per spike (TSN), and FSN was performed, and subsequently, a major QTL on chromosome 3DL was further validated in different genetic backgrounds.

## Materials and Methods

### Plant Materials and Field Trials

Quantitative trait locus mapping was conducted using a population of 187 DH lines derived from a cross of K1 × CM42 (K1/CM42). CM42, kindly provided by Prof. Wuyun Yang (Sichuan Academy of Agricultural Science, China), is a backbone parent with excellent agronomic traits such as high TKW and high yield (Yang et al., 2009). K1, bred by our lab, is characterized by high spikelet number per spike and GNS. Two populations, including a recombinant inbred line (RIL) populations derived from a cross of K1 × Kechengmai4 (K1/K4, 70 F_7_ RILs) and a F_2_ populations derived from a cross of K1 × Yangfumai2 (K1/YFM2, 75 F_2_ lines), were employed to further validate the major QTL.

The K1/CM42 lines along with parents were grown in five replicates following randomized complete-block design at the following two experimental sites: Shuangliu of Sichuan province (103°52′E, 30°34′N) and Shifang of Sichuan province (104°11′E, 31°6’N), China. The field trials were carried out in two crop seasons of 2017–18 and 2018–19 at Shuangliu, and three crop seasons of 2016–17, 2017–18, and 2018–19 at Shifang. Each plot consisted of five 200-cm rows with an inter-row spacing of 20 cm. The sowing density was 50 seeds per row. The K1/K4 lines were generated by single-seed descent and cropped at Shuangliu and Shifang in 2018–19 crop seasons. Each plot consisted of two 200-cm rows with an inter-row spacing of 20 cm. The K1/YFM2 population was cropped at Shuangliu in 2019–20 crop seasons in 200-cm rows with 15-cm space between individuals.

### Phenotypic Evaluation and Statistical Analysis

The TSN, FSN, plant height (PH), and SL of the K1/CM42 and K1/K4 populations were manually measured at maturity by using plants from middle row of each plot. For the K1/CM42 population, the primary spike of 10 random plants from each family in 2017–18 and 2018–19 crop seasons and three random plants from each family in 2016–17 crop seasons were selected. For the K1/K4 population, the primary spikes of five random plants from each family were selected. For the K1/YFM2 population, the TSN, FSN, PH, and SL of primary spike of each individual were measured. Subsequently, all selected spikes were harvested and manually threshed for evaluating GNS, TKW, grain width (GW), and grain length (GL) using SC-G software (WSeen, Hangzhou, China).

The frequency distribution of traits in each environment and their correlation were calculated using SPSS version 20.0 for Windows (IBM SPSS, Armonk, NY, United States). The best linear unbiased predictions (BLUPs), which were used for QTL detection, correlation analyses, and effect analyses, were calculated using the R package “lme4.” Analysis of variance (ANOVA) was performed using the data from 2017–18 and 2018–19 crop seasons in the K1/CM42 population by SPSS. The broad-sense heritability (*H*^2^) was calculated according to the method described by Smith et al. (1998) and Muqaddasi et al. (2019). Student’s *t* test used to evaluate the significance of difference was performed by SPSS.

### Genotyping

Genomic DNA of each line in the K1/CM42 population and their parents was extracted using the 2 × cetyl trimethylammonium bromide (CTAB) method and hybridized on the Wheat 55K SNP array by China Golden Marker (Beijing, China). DNA integrity was checked and confirmed on agarose gels, and DNA quantity was measured by spectrophotometry.

### Genetic Map Construction and Quantitative Trait Locus Detection

IciMapping 4.1 (Meng et al., 2015) and JoinMap 4.1 were used for genetic construction and QTL detection in the present study. First, the function of “bin” in IciMapping 4.1 was used to remove redundant markers based on their segregation patterns in the mapping population with the parameters of “Missing Rates” and “Distortion Value” being set as 20% and 0.01, respectively. Then, the function of “Population” in JoinMap 4.1 was used to create groups with limit of detection (LOD) score values ranging from 2 to 10. Finally, the Kosambi mapping function was used to order the bin markers with the parameters being set as LOD ≥ 5 and round = 3 in JoinMap 4.1. To reduce the complexity of QTL mapping analyses, only one marker was selected as a delegate from each bin to construct the linkage map. QTL detection in each environment was performed by IciMapping 4.1 with the inclusive composite interval mapping (ICIM), and a test of 1,000 permutations was used to identify the LOD threshold that corresponded to a genome-wide false discovery rate of 5% (*P* < 0.05). The missing phenotype was deleted in QTL analysis.

### Development of Kompetitive Allele-Specific PCR Markers

Probe DNA sequences for selected SNP were subjected to design Kompetitive Allele-Specific PCR (KASP) markers using the Triticeae Multi-omics Center^1^. Sequence probes for the FAM signal and the HEX signal were added to the primers of two parental genotypes, respectively. The KASP assays were performed in Bio-Rad CFX96 real-time PCR system with 10-μl reaction volumes consisting of 5 μl of 2 × master mix, 0.2 μl of primer mix, 3 μl of ddH_2_O, and 2 μl of DNA sample (50–150 ng/μl). Thermal cycling conditions were 94°C for 15 min hot-start activation, followed by a touchdown phase of 10 cycles (94°C for 20 s, touchdown at 61°C initially and then decreased by 0.6°C per cycle for 60 s) and finally 26 cycles of regular PCR (94°C for 20 s, 55°C for 60 s, and rest plates at 37°C for 1 min). Further cycling and resting were performed if the clustering is not significant: 94°C for 20 s, followed by 57°C for 60 s (3–10 cycles per step). The information of DNA sequences flanking the SNP was kindly provided by Prof. Jizeng Jia’s Lab (Institute of Crop Sciences, Chinese Academy of Agricultural Sciences).

### Prediction of Candidate Gene

The physical positions of flanking markers were obtained by blasting against (*E*-value of 1e-10) the genome assembly of Chinese Spring (CS)^2^. Genes among the mapping interval were extracted from IWGSC RefSeq v1.1 annotation^3^. The annotations and functions of a given gene were analyzed using UniProt^4^. Gene collinearity among wheat, barley, rice, and maize was performed using the Triticeae-Gene Tribe^5^. The temporal and spatial gene expression patterns were extracted from Wheat Expression Browser^6^. To further explore the potential candidate genes, we designed the corresponding specific primers^1,7^ to isolate and sequence these candidate genes (Tsingke Biotech Co., Ltd., China). The primer sequences were listed in Supplementary Table 2. Twenty-five-microliter PCR reaction volumes were as follows: 12.5 μl of 2 × EasyTaq PCR SuperMix (TransGen Biotech Co., Ltd., China), 3 μl of DNA sample, 1 μl of primer mix, and 7.5 μl of ddH_2_O.

## Results

### Phenotypic Variation

**FIGURE 1:**
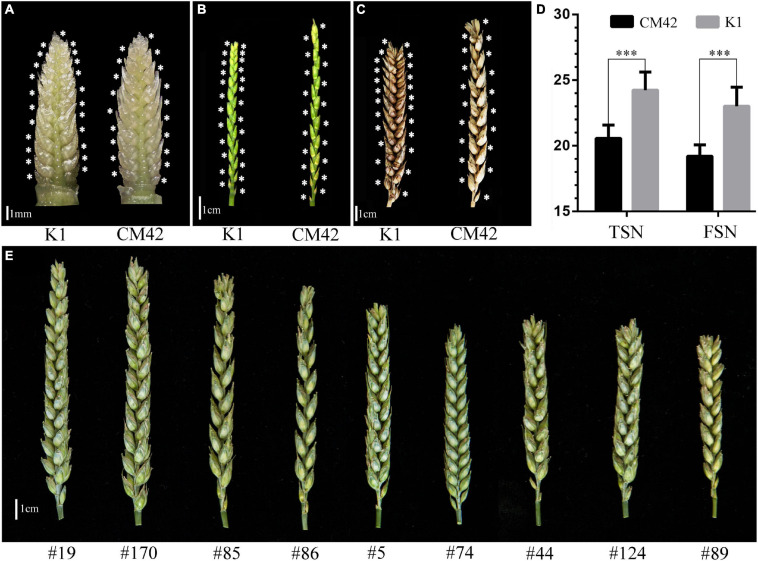
Spike morphology of the parents and representative lines. The morphology of spikes of K1 and CM42 at young spike stage when terminal spikelet appeared **(A)**, heading stage (awn was cut) **(B)**, and maturity (awn was cut) **(C)** are shown. **(D)** The mean of total spikelet number per spike (TSN) and fertile spikelet number per spike (FSN) of K1 and CM42 at maturity in five environments. **(E)** The morphology of spikes of representative lines from the K1/CM42 population; ^∗∗∗^ represents significance at *P* < 0.001.

**FIGURE 2:**
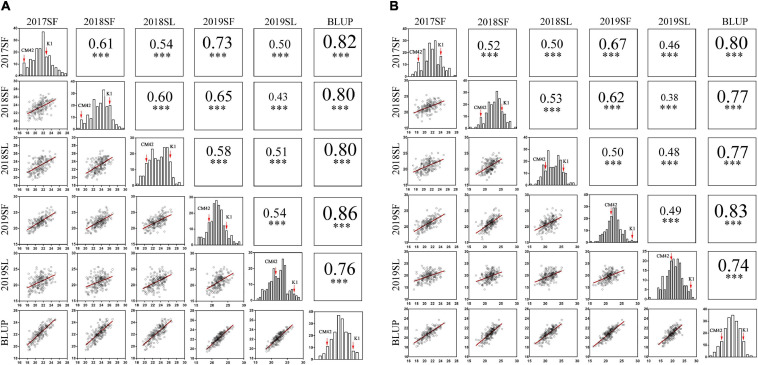
Phenotypic performances, distribution, and correlation coefficients for total spikelet number per spike (TSN) **(A)** and fertile spikelet number per spike (FSN) **(B)** of parents and K1/CM42 lines in five environments and their corresponding best linear unbiased prediction (BLUP) values; ^∗∗∗^ represents significance at *P* < 0.001.

Significant differences (*P* < 0.001) on TSN and FSN between K1 and CM42 across five environments and their corresponding BLUP data were detected. K1 showed more TSN and FSN than did parent CM42 (Figure 1 and Table 1). In addition, we observed the development of spikelet, and we found that K1 showed more TSN at a very early stage of spike development than that of CM42, as well as at heading and maturity stages, indicating that spikelet number is determined at an early stage of wheat reproductive development (Figure 1). For the K1/CM42 lines, the TSN and FSN ranged from 16.3 to 26.8 and from 14.2 to 25.8, respectively, showing wide and significant variation and approximately normal distribution (Table 1 and Figure 2). In addition, the TSN and FSN values across all environments and BLUP data were significantly positively correlated (*P* < 0.001), showing high broad-sense heritabilities (0.85 for TSN and 0.82 for FSN), indicating that they were mainly under genetic control (Table 1).

**TABLE 1:** Phenotypic variation and heritability (*H*^2^) of TSN and FSN for the parents and the K1/CM42 lines in different environments.

Trait	Environment	Parents		The K1/CM42 lines								*H*^2^
		K1	CM42	Range	Min.	Max.	Mean	SD	CV (%)	SK.	Ku.	
TSN	2017SF	21.8 ± 0.84	18.6 ± 1.14^∗∗∗^	7	18	25	21.21	1.478	6.97%	0.116	–0.239	0.85
	2018SF	25.2 ± 1.32	20.7 ± 1.64^∗∗∗^	6.7	20.1	26.8	23.42	1.405	6.00%	–0.17	–0.46	
	2018SL	25.2 ± 0.63	21 ± 0.82^∗∗∗^	7.2	19.5	26.7	23.02	1.511	6.56%	–0.108	–0.779	
	2019SF	25.2 ± 0.42	21.7 ± 1.16^∗∗∗^	8.3	18.3	26.6	22.05	1.543	7.00%	0.115	0.391	
	2019SL	24.2 ± 1.23	20.6 ± 0.84^∗∗∗^	9.4	16.3	25.7	21.01	1.878	8.94%	–0.029	–0.287	
	BLUP	23.7	20.7	5.3	19.4	24.7	22.14	1.087	4.91%	–0.2	–0.3	
FSN	2017SF	21 ± 0.71	18.2 ± 1.3^∗∗∗^	7.7	17.3	25	20.91	1.515	7.24%	0.081	–0.29	0.82
	2018SF	22.8 ± 1.62	18.5 ± 1.65^∗∗∗^	8.2	17.2	25.4	21.61	1.439	6.66%	–0.2	0.04	
	2018SL	23.3 ± 0.95	19.4 ± 0.7^∗∗∗^	8.4	16.7	25.1	21.02	1.601	7.62%	0.008	–0.566	
	2019SF	25.2 ± 0.42	20.7 ± 1.16^∗∗∗^	9.7	16.1	25.8	21.04	1.608	7.64%	0.042	0.579	
	2019SL	23.4 ± 1.71	18.9 ± 1.1^∗∗∗^	9.9	14.2	24.1	19.66	1.862	9.47%	–0.104	–0.246	
	BLUP	22.4	19.7	5.5	18.2	23.7	20.85	1.052	5.04%	–0.19	-0.25	

*Note. BLUP, best linear unbiased prediction; SD, standard deviation; CV, coefficient of variation; SK., skewness; Ku., kurtosis; H^2^, broad-sense heritability. ^∗∗∗^ represents significance at *P* < 0.001.*

Phenotypic correlations between TSN, FSN, and other yield related traits are listed in Table 2. Significant and positive correlations (*P* < 0.001) between TSN and FSN and GNS, and significant and negative correlation (*P* < 0.01) between TSN and GL, and FSN and GL were observed in the K1/CM42 population. The highest positive correlation was detected between TSN and FSN (*r* = 0.96). No significant correlation was detected between TSN and TKW, TSN and PH, TSN and SL, FSN and TKW, FSN and PH, and FSN and SL were observed in the K1/CM42 population.

**TABLE 2:** Correlation coefficients for TSN and FSN with other agronomic traits.

Traits	TSN	FSN
FSN	0.96^∗∗∗^	
GNS	0.57^∗∗∗^	0.63^∗∗∗^
PH	0.1	0.08
SL	0.05	0.04
TKW	−0.11	−0.13
GW	0.04	0.05
GL	−0.23^∗∗^	−0.21^∗∗^

*Note. The correlation coefficients were evaluated by the BLUP data. TSN, total spikelet number per spike; FSN, fertile spikelet number per spike; GNS, grain number per spike; PH, plant height; SL, spike length; TKW, thousand kernel weight; GW, grain width; GL, grain length; BLUP, best linear unbiased prediction. ^∗∗^ and ^∗∗∗^ represent significance at *P* < 0.01 and *P* < 0.001, respectively.*

### Linkage Map Construction

Among 53,063 SNPs from the Wheat 55K SNP array, 14,645 SNP markers (27.6%) were polymorphic between the two parental lines. Of them, 822 SNPs had more than 20% missing data points, 10 SNPs were not anchored on the physical linkage map, and 745 SNPs were not anchored on the genetic linkage map. Finally, the rest 13,068 SNP markers (call rate ≥ 80%) were used for linkage analysis and map construction. These markers were divided into 2,406 bins: 1,384 bins contain one SNP marker only, 1,022 bins contain multiple SNPs, and the largest bin contains 626 SNP markers on 4B. Linkage analysis showed that 2,406 bin markers were mapped on 27 linkage maps for the 21 chromosomes of wheat, in which two linkage groups were constructed for each of chromosomes 1A, 1D, 5A, 5B, 6A, and 6D. Based on the genetic information, all SNP markers including bin markers and redundant markers were integrated onto the genetic map with a total length of 3,091.39 cM and an average interval distance of 1.28 cM per bin and 0.24 cM per marker. All of the mapped markers were located to the A (33.75%), B (45.98%), and D (20.26%) genomes with a total length of 1,009.07, 895.54, and 1,186.79 cM, respectively. In addition, the lengths of constructed linkage maps range from 17.67 (1A-1) to 206.47 cM (5D); the markers on linkage maps range from 20 (1D-2) to 1228 (4B); the average interval distance between adjacent markers ranges from 0.08 (4B) to 4.37 cM (1D-2); and the average interval distance between adjacent bin markers ranges from 0.63 (2B) to 5.14 cM (1D-2) (Table 3 and Supplementary Table 1). Based on the physical locations of these SNPs on the CS genome, the marker order was relatively consistent with that in the wheat genome assembly on most of the chromosomes (1A, 1B, 2A, 3B, 3D, 4A, 4B, 5A, 5B,5D, 6A, 6D, 7A, 7B, and 7D) (Supplementary Figure 1).

**TABLE 3:** General information of the high-density genetic linkage map.

Chromosome	Group	Number of bin markers	Number of markers	Length (cM)	Density (cM/bin)	Density (cM/marker)
1A	1	21	91	17.67	0.84	0.19
	2	65	744	68.84	1.06	0.09
1B	1	167	752	132.77	0.80	0.18
1D	1	17	20	87.43	5.14	4.37
	2	80	688	125.60	1.57	0.18
2A	1	101	305	145.13	1.44	0.48
2B	1	159	631	100.80	0.63	0.16
2D	1	81	302	140.51	1.73	0.47
3A	1	149	660	102.83	0.69	0.16
3B	1	98	989	90.43	0.92	0.09
3D	1	80	575	178.38	2.23	0.31
4A	1	142	889	194.68	1.37	0.22
4B	1	103	1,228	98.91	0.96	0.08
4D	1	86	271	107.92	1.25	0.40
5A	1	163	795	154.26	0.95	0.19
	2	17	40	46.85	2.76	1.17
5B	1	69	723	75.19	1.09	0.10
	2	96	310	92.56	0.96	0.30
5D	1	95	258	206.47	2.17	0.80
6A	1	47	328	71.71	1.53	0.22
	2	20	48	26.78	1.34	0.56
6B	1	144	693	114.38	0.79	0.17
6D	1	32	122	77.24	2.41	0.63
	2	29	127	94.25	3.25	0.74
7A	1	139	511	180.32	1.30	0.35
7B	1	141	683	190.50	1.35	0.28
7D	1	65	285	168.97	2.60	0.59
A genome	10	864	4,411	1,009.07	1.17	0.23
B genome	8	977	6,009	895.54	0.92	0.15
D genome	9	565	2,648	1,186.79	2.10	0.45
Total	27	2,406	13,068	3,091.39	1.28	0.24

### Quantitative Trait Locus Mapping

Phenotypic data of TSN and FSN evaluated in five environments and their corresponding BLUP values were used for QTL mapping, where the BLUP values were treated as an additional environment. A total of 27 QTLs for TSN and FSN were detected, which were located on chromosomes 1B, 1D, 2B, 2D, 3D, 4A, 4D, 5A, 5B, 5D, 6A, 6B, and 7D, respectively. Among them, five QTLs defined as stable were detected in multiple environments and combined QTL analysis, explaining the phenotypic variance ranging from 3.64% to 23.28%. The rest QTLs were detected in one environment, explaining 2.47–18.4% of the phenotypic variance (Table 4).

**TABLE 4:** Summary of QTLs detected in the K1/CM42 population.

Trait	QTL	Env.	Chr.	Pos. (cM)	Interval (cM)	Flanking markers	LOD	PVE (%)	Add	Physical pos. (Mb)
TSN	*QTsn.cib-1B.1*	2019SL	1B	82.8	82.78*–*83.11	AX-111070175*–*AX-110565341	14.95	15.04	–0.98	584.32*–*591.96
	*QTsn.cib-1B.2*	2019SL	1B	89.3	88.25*–*89.43	AX-109977434*–*AX-109971635	8.53	8.19	0.73	630.07*–*632.63
	*QTsn.cib-1D*	2017SF	1D	88.1	87.67*–*88.23	AX-109917997*–*AX-108878320	4.1	5.27	–0.3	432.71*–*433.18
	*QTsn.cib-2B.1*	2019SF	2B	1.7	1.26*–*1.86	AX-109825368*–*AX-110387848	3.87	4.11	–0.32	158.35*–*158.35
	*QTsn.cib-2D*	2018SF	2D	98	94.3*–*100.31	AX-89629279*–*AX-112286381	9.03	4.24	–0.45	535.22*–*648.11
		2018SL		94.4	94.3*–*100.31	AX-89629279*–*AX-112286381	3.89	10.23	–0.5	
		BLUP		100.2	94.3*–*100.31	AX-89629279*–*AX-112286381	9.79	3.64	–0.38	
	*QTsn.cib-3D*	2017SF	3D	104.5	103.64*–*108.09	AX-110914105*–*AX-109429351	13.63	18.93	–0.58	549.49*–*555.21
		2018SL		105.4	103.64*–*108.09	AX-110914105*–*AX-109429351	6.1	12.01	–0.5	
		2019SF		104.5	103.64*–*108.09	AX-110914105*–*AX-109429351	18.51	23.15	–0.76	
		2019SL		103.7	103.64*–*108.09	AX-110914105*–*AX-109429351	7.49	6.79	–0.66	
		BLUP		104.5	103.64*–*108.09	AX-110914105*–*AX-109429351	17.79	6.5	–0.48	
	*QTsn.cib-5A*	2019SF	5A	152	103.64*–*154.26	AX-111472310*–*AX-108732747	7.82	8.52	0.46	573.56*–*597.74
	*QTsn.cib-5B*	2018SF	5B	25.1	25.05*–*31.04	AX-110050262*–*AX-108791526	6.06	2.47	0.33	10.13*–*34.49
	*QTsn.cib-5B*	2018SF	5B	0.6	0.56*–*0.89	AX-108863479*–*AX-109820694	7.96	3.27	–0.38	565.01*–*565.98
	*QTsn.cib-5D*	2018SL	5D	14.8	14.75*–*24.21	AX-110472882*–*AX-110213253	4.17	7.59	–0.4	28.74*–*157.2
	*QTsn.cib-5D*	2017SF	5D	33	32.78*–*33.01	AX-110186027*–*AX-111496275	8.3	10.75	–0.43	279.12*–*284.12
	*QTsn.cib-6A*	2018SF	6A	2.9	2.63*–*3.34	AX-109340483*–*AX-108959026	9.2	3.97	–0.42	52.98*–*97.59
	*QTsn.cib-6B*	2017SF	6B	62	61.84*–*62.4	AX-110602749*–*AX-109036922	5.4	7.87	0.37	100.68*–*127.16
	*QTsn.cib-7D*	2019SF	7D	79.5	78.81*–*79.93	AX-111061288*–*AX-110826147	11.39	13.21	–0.6	65.5*–*66.54
FSN	*QFsn.cib-1B.1*	2018SL	1B	52.3	52.24*–*52.57	AX-110365753*–*AX-110547478	5.52	10.4	–0.5	251.53*–*347.65
	*QFsn.cib-1B.2*	2019SL	1B	82.8	82.76*–*83.11	AX-111070175*–*AX-110565341	15.16	15.57	–1.05	584.32*–*591.96
	*QFsn.cib-1B.3*	2019SL	1B	89.4	88.25*–*89.43	AX-109977434*–*AX-109971635	8.47	8.01	0.76	630.07*–*632.63
	*QFsn.cib-1D*	2018SF	1D	85.3	83.84*–*85.3	AX-108813568*–*AX-111890874	7.39	6.51	–0.46	436.08*–*436.2
	*QFsn.cib-2D*	2019SF	2D	75.3	74.01*–*75.49	AX-110390887*–*AX-111601893	5.66	4.42	–0.41	16.85*–*23.3
	*QFsn.cib-3D*	2017SF	3D	104.4	103.64*–*108.09	AX-110914105*–*AX-109429351	11.2	23.28	–0.65	549.49*–*555.21
		2018SL		105.3	103.64*–*108.09	AX-110914105*–*AX-109429351	3.73	7.23	–0.41	
		2019SF		103.7	103.64*–*108.09	AX-110914105*–*AX-109429351	14.94	10.8	–0.64	
		2019SL		103.6	103.64*–*108.09	AX-110914105*–*AX-109429351	6.53	5.97	–0.65	
		BLUP		103.7	103.64*–*108.09	AX-110914105*–*AX-109429351	12.52	11.52	–0.39	
	*QFsn.cib-4A.1*	BLUP	4A	98	97.33*–*98.03	AX-109508535*–*AX-109957052	18.34	18.26	–0.5	603.5*–*605.63
	*QFsn.cib-4A.2*	BLUP	4A	108.7	108.3*–*113.43	AX-110042237*–*AX-110488353	10.44	9.74	0.36	606.51-612.07
	*QFsn.cib-4D*	BLUP	4D	34.4	31.49*–*34.44	AX-110984743*–*AX-110458818	6.68	5.74	0.29	9.31*–*9.76
	*QFsn.cib-5A*	2018SF	5A	71.8	71.77*–*74.49	AX-110012348*–*AX-111591583	18.09	18.4	0.77	452.12*–*459.97
		2019SF		74.1	71.77*–*74.49	AX-110012348*–*AX-111591583	8.26	5.86	0.48	
	*QFsn.cib-5B*	2018SF	5B	0.6	0.56*–*0.89	AX-108863479*–*AX-109820694	7.05	6.21	–0.46	565.01*–*565.98
	*QFsn.cib-5D*	2017SF	5D	32.9	32.78*–*33.01	AX-110186027*–*AX-111496275	3.9	7.57	–0.37	279.12*–*284.12
	*QFsn.cib-7D*	2017SF	7D	78.8	76.18*–*88.52	AX-108882010*–*AX-89366204	3.96	7.51	–0.39	63.48*–*90.62
		2019SF		80	76.18*–*88.52	AX-108882010*–*AX-89366204	16.74	12.58	–0.72	
		BLUP		79.1	76.18*–*88.52	AX-108882010*–*AX-89366204	10.74	9.87	–0.38	

*Note. Add, additive effect (positive values indicate that alleles from CM42 are increasing the trait scores, and negative values indicate that alleles from K1 are increasing the trait scores); Chr., chromosome; Pos., position; PVE, phenotypic variation explained; LOD, limit of detection.*

For TSN, 14 QTLs were detected on chromosomes 1B, 1D, 2B, 2D, 3D, 5A, 5B, 5D, 6A, 6B, and 7D. Of them, a major QTL *QTsn.cib-3D* was stably detected in four environments and combined QTL analysis, explaining 6.5% to 23.15% of the phenotypic variance with LOD values ranging from 6.1 to 18.51. Another stable QTL *QTsn.cib-2D* was detected in two environments and combined QTL analysis, explaining 3.64–10.23% of the phenotypic variance. The positive alleles of the two loci were all contributed by K1. For the remaining 12 QTLs, they were detected in one environment and explained 2.47–15.04% of the phenotypic variance. The positive alleles of eight loci were from K1, and four loci were from CM42 (Table 4).

For FSN, 13 QTLs were identified on chromosomes 1B, 1D, 2D, 3D, 4A, 4D, 5A, 5B, 5D, and 7D. Of them, *QFsn.cib-3D* was a major QTL and stably detected in four environments and combined QTL analysis, explaining 5.97–23.28% of the phenotypic variance with LOD values ranging from 3.73 to 14.94. *QFsn.cib-7D* was detected in two environments and combined QTL analysis, explaining 7.51–12.58% of the phenotypic variance. The positive alleles of the two loci were contributed by K1. *QFsn.cib-5A* was detected in two environments with the phenotypic variance ranging from 5.86% to 18.4%, and the positive allele was contributed by CM42. The rest of the 10 QTLs were detected in single environment, accounting for 4.42–18.26% of the phenotypic variance. The positive alleles of seven and three loci were contributed by K1 and CM42, respectively (Table 4).

Remarkably, a comparison of the intervals of loci for TSN and FSN revealed that *QTsn.cib-3D* and *QFSN.cib-3D* were co-located in the interval of *AX-110914105–AX-109429351.* We thus temporarily designated the locus as *QTsn/Fsn.cib-3D* (Table 4).

### Effects of *QTsn/Fsn.cib-3D* on Total Spikelet Number Per Spike, Fertile Spikelet Number Per Spike, and Yield-Related Traits in the K1/CM42 Population

**FIGURE 3:**
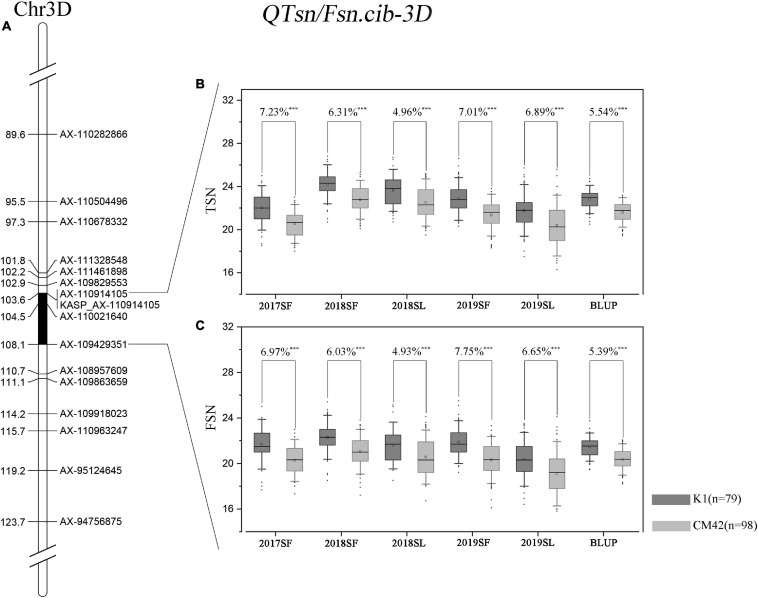
Genetic map of the major quantitative trait locus (QTL) *QTsn/Fsn.cib-3D* and its effect. Genetic map integrated with the developed Kompetitive Allele-Specific PCR (KASP) marker *KASP_AX-110914105* **(A)**. The black area is the interval of the major QTL *QTsn/Fsn.cib-3D* **(A)**. Effects of the major QTL *QTsn/Fsn.cib-3D* on total spikelet number per spike (TSN) **(B)** and fertile spikelet number per spike (FSN) **(C)** shown as box plots calculated after grouping the K1/CM42 population into two classes based on the KASP marker; K1 and CM42 indicate the lines with and without positive alleles of *QTsn/Fsn.cib-3D*; ^∗∗∗^ represents significance at *P* < 0.001.

**FIGURE 4:**
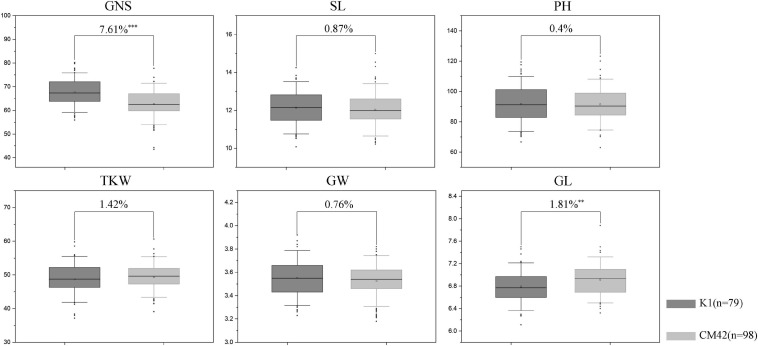
Effects of *QTsn/Fsn.cib-3D* on grain number per spike (GNS), spike length (SL), plant height (PH), thousand kernel weight (TKW), grain width (GW), and grain length (GL) in the K1/CM42 population. K1 and CM42 indicate the lines with and without positive alleles of *QTsn/Fsn.cib-3D*; ^∗∗^ and ^∗∗∗^ represent significance at *P* < 0.01 and *P* < 0.001, respectively.

*QTsn/Fsn.cib-3D* was a major and stable QTL influencing TSN and FSN. We subsequently developed a KASP marker (*KASP_AX-110914105*) based on SNP of the flanking marker (*AX-110914105*) (Supplementary Table 2). As expected, on the basis of the genotyping results for *KASP_AX-110914105* in the K1/CM42 population, significant differences (*P* < 0.001) on TSN and FSN were detected between lines with homozygous alleles from K1 and CM42 in each environment and BLUP data (Figure 3). In addition, we further analyzed the effects of *QTsn/Fsn.cib-3D* on yield-related traits in the K1/CM42 population. The results revealed that *QTsn/Fsn.cib-3D* significantly affected GNS (7.61%) and GL (1.81%) but did not influence the TKW, PH, SL, and GW (Figure 4).

### Validation of *QTsn/Fsn.cib-3D* in Different Genetic Backgrounds

**FIGURE 5:**
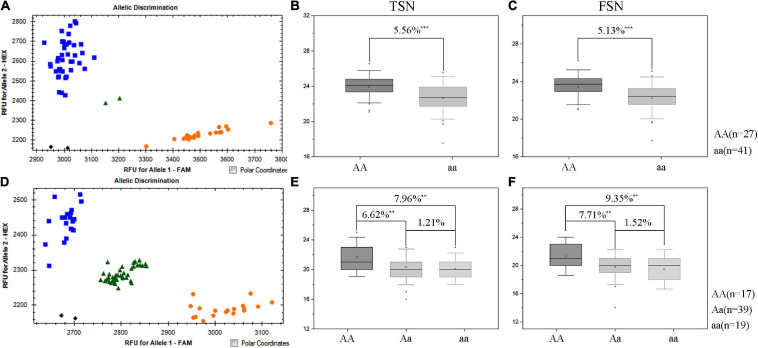
Validation of *QTsn/Fsn.cib-3D* in two populations with different genetic environments. Fluorescence PCR typing results of the Kompetitive Allele-Specifc PCR (KASP) marker *KASP_AX-110914105* in K1/K4 **(A)** and K1/YFM2 **(D)** populations. Orange circle represents lines carrying the allele of K1; blue box represents lines with the allele of K4 **(A)** and YFM2 **(D)**, respectively. Effects of *QTsn/Fsn.cib-3D* on total spikelet number per spike (TSN) **(B)** and fertile spikelet number per spike (FSN) **(C)** in the K1/K4 population. Effects of *QTsn/Fsn.cib-3D* on total spikelet number per spike (TSN) **(E)** and fertile spikelet number per spike (FSN) **(F)** in the K1/YFM2 population; ^∗∗^ and ^∗∗∗^ represent significance at *P* < 0.01 and *P* < 0.001, respectively; AA represents the homozygous allele from K1; aa represents the homozygous allele from non-K1; Aa represents the heterozygous allele.

Since the favorable allele of *QTsn/Fsn.cib-3D* was from K1, the other two populations (K1/K4 and K1/YFM2) containing a common parent K1 were used to validate the effects of *QTsn/Fsn.cib-3D* in different genetic backgrounds. The KASP marker *KASP_AX-110914105* closely linked to *QTsn/Fsn.cib-3D* was used to assess the alleles from the common parent K1. For the K1/K4 population, 27 lines were found to be homozygous for the K1 allele, and the remaining 41 lines were found to be homozygous for the non-K1 allele (Figure 5). For the K1/YFM2 population, 17 plants were found to be homozygous for the K1 allele, 19 plants were found to be homozygous for the non-K1 allele, and the remaining 39 plants were found to be heterozygous allele (Figure 5). Subsequently, Student’s *t*-test was performed to analyze significant differences on TSN and FSN (*P* < 0.05) between classes with different allele. As expected, the lines with the homozygous K1 allele had significantly (*P* < 0.01) higher TSN (5.56–7.96%) and FSN (5.13–9.35%) than those carrying the non-K1 homozygous allele (Figure 5). Moreover, a significant difference on TSN (6.62%) and FSN (7.71%) was also detected between lines with K1 homozygous allele and heterozygous allele (Figure 5).

### Candidate Genes Analysis of *QTsn/Fsn.cib-3D*

**FIGURE 6:**
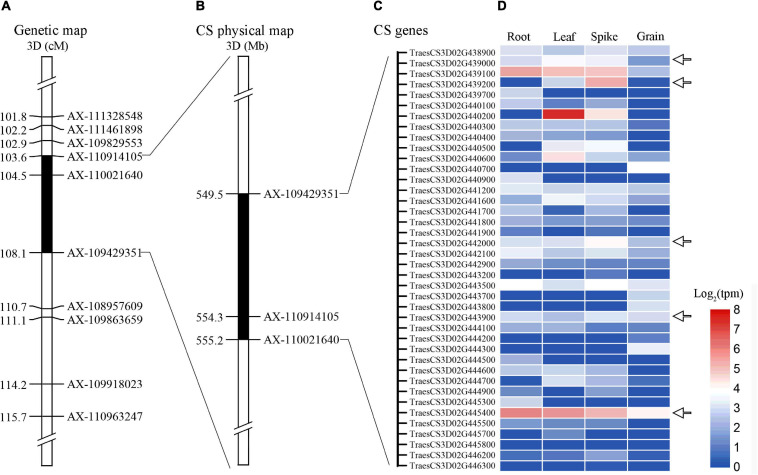
Expression patterns of genes in the physical interval of *QTsn/Fsn.cib-3D* in various tissues. Black areas are the genetic interval **(A)** and physical interval of *QTsn/Fsn.cib-3D* on chromosome 3D **(B)**; **(C)** and **(D)** represent the genes that were expressed in various tissues and their expression patterns.

**FIGURE 7:**
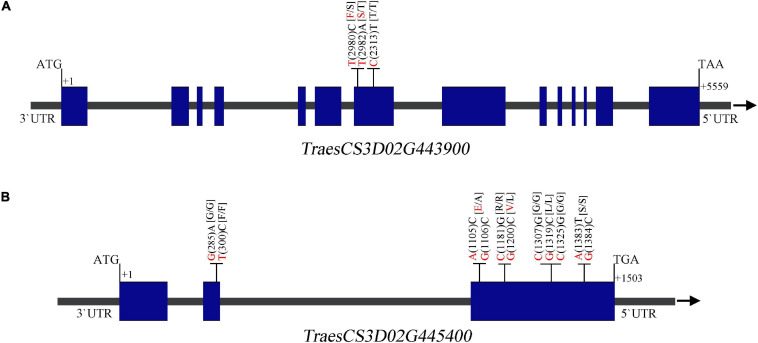
Sequence analysis of the *TraesCS3D02G443900* **(A)** and *TraesCS3D02G445400* **(B)** showing the single-nucleotide polymorphisms (SNPs) and corresponding amino acid variations between K1 and CM42, and K4 and YFM2. The nucleotide of K1 and the other parents is shown in red and black, respectively. Blue box and gray line on gene structure schematic diagrams represent the exome and intron or UTR, respectively.

Alignment of the flanking markers of *QTsn/Fsn.cib-3D* showed that it was corresponding to a physical interval of 549.49–555.2 Mb on chromosome arm 3DL (Figure 6 and Table 4). According to CS reference genome, there were 76 predicated genes in this interval (Figure 6). An analysis of the spatial expression patterns (Borrill et al., 2016; Ramírez-González et al., 2018) showed that 40 genes were expressed in various tissues, and several of them were abundantly expressed in spikes, indicating that they are supposed to be involved in spike development (Figure 6). Among these genes, *TraesCS3D02G439000*, *TraesCS3D02G439200*, *TraesCS3D02G442000*, *TraesCS3D02G443900*, and *TraesCS3D02G44540*0 are likely associated with spike growth and development according to the gene annotation (Supplementary Table 3). To further analyze potential polymorphism of these candidate genes between K1 and CM42, and K4 and YFM2, we designed gene-specific primers to isolate and resequence the five genes (Supplementary Table 2). For *TraesCS3D02G439000*, *TraesCS3D02G439200*, and *TraesCS3D02G442000*, no sequence variation in coding sequences (cds) between K1 and the other three parents was detected (Supplementary Figures 4–6). For *TraesCS3D02G443900*, three SNPs in the cds between K1 and the other three parents were detected, and two of them were non-synonymous SNPs (Figure 7 and Supplementary Figure 7). Interestingly, we isolated and assembled the complete coding sequences of *TraesCS3D02G44540*0 from K1 and YFM2 using these designed specific primers. However, only partial coding sequences could be obtained from CM42 and K4 (Supplementary Figure 8). We speculated that significant differences may exist in the sequences of *TraesCS3D02G44540*0 between K1 and CM42 and K4. Based on these sequences, two synonymous SNPs in the coding sequence between K1 and the other three parents, and extra six synonymous SNPs and three non-synonymous SNPs between K1 and YFM2 were detected, respectively (Figure 7 and Supplementary Figure 8).

## Discussion

Grain number per spike as one of three major components determining wheat yield is ultimately determined by the number of FSN and fertile floret per spikelet. In the present study, by assessing two spikelet number-related traits TSN and FSN in a DH population among five environments, we found that TSN and FSN were significantly and positively correlated with GNS (Table 2) with a high heritability (0.85 for TSN and 0.82 for FSN) (Table 1). The results were consistent with previous knowledge (Che et al., 2018; Würschum et al., 2018; Zhang et al., 2019) that they are environmentally stable yield components and mainly determined by genetic factors. Therefore, increasing spikelet number could be considered as an effective strategy for increasing GNS and hence the grain yield.

### Comparison of Constructed Genetic Linkage With Previous Studies

Quantitative trait locus analysis is a well-established and widely used tool for dissecting the genetic basis of complex traits, and a high-density genetic map plays a fundamental role in QTL analysis (Salvi and Tuberosa, 2005; Cooper et al., 2009; Yamamoto et al., 2009). In the present study, by using the wheat 55K SNP array, a high-density genetic map containing 13,068 SNPs with a total length of 3,091.39 cM was constructed. The marker order was relatively consistent with that in the wheat genome assembly on most of the chromosomes (Supplementary Table 1 and Supplementary Figure 1). It is well known that the SNP markers in the Wheat 55K SNP array were well chosen from the 660K SNP array. Compared with previously reported genetic maps, we extracted 2,739 and 4,860 common mapped markers from the Wheat 55K and 660K SNP array, respectively (Supplementary Table 4) (Cui et al., 2017; Liu et al., 2018a). The genetic positions of these markers were relatively consistent in the three genetic maps (Supplementary Figures 2, 3), indicating that the genetic map constructed in present study was accurate and credible and could be effectively used for QTL analysis.

### Comparison of Stable Quantitative Trait Loci for Spikelet Number With Previous Studies

Over the past decades, QTLs that control spikelet number have been extensively studied and identified on almost every wheat chromosome. In this study, five stable QTLs for TSN and FSN were detected on chromosomes 2D, 3D, 5A, and 7D. Of them, *QTsn/Fsn.cib-3D* associated with TSN and FSN was detected as a major QTL for spikelet number. This QTL was mapped in a 4.455-cM interval and physically corresponding to 549.49 and 555.2 Mb on chromosome arm 3DL according to the physical location of flanking markers (Table 4 and Figure 3). As far as we know, several QTLs for spikelet number on chromosome 3D including *QSns.sau-3D*, *QSsn.czm-3D*, *QSspn.cau-3D*, and *QSpn.ipk-3D* had been reported in previous studies. *QSsn.czm-3D* and *QSspn.cau-3D* were located on the short arm of chromosome 3D flanked by *3DS_2577014_1698-IWA4559* and *Xcau.3D-5-Xcau.3D-6*, respectively (Cui et al., 2012; Chen et al., 2020). *QSns.sau-3D* detected by Luo et al. (2016) was located between *gpw3109* (306.5 Mb) and *gdm8* (357.1 Mb). Given that the physical intervals of these QTLs were different from those of the *QTsn/Fsn.cib-3D*, the *QTsn/Fsn.cib-3D* may be different from these loci. The remaining QTL, *QSpn.ipk-3D*, detected by Pestsova et al. (2006) was located between *Xgwm383* (532.19 Mb) and *Xgwm3* (579.89 Mb). Although the QTL seems overlapped with *QTsn/Fsn.cib-3D*, it was a minor QTL detected in one environment, explaining only 3.9% of the phenotypic variance. These results indicated that *QTsn/Fsn.cib-3D* is probably a novel and major QTL for spikelet number. Additionally, the rest three stable QTLs *QFsn.cib-7D*, *QTsn.cib-2D*, and *QFsn.cib-5A* in present study were overlapped or close to QTLs for spikelet number reported previously, indicating they are likely alleles. For example, *QFsn.cib-7D* was located between 66.54 and 90.62 Mb on chromosome arm 7DS. This interval was overlapped with *QTspn.cau-7D* and *QFspn.cau-7D* reported by Chen et al. (2020). *QTsn.cib-2D* mapped in the physical interval of 534.32–648.11 Mb on chromosome arm 2DL was overlapped with *QTssn.WJ.2D.2* and *QFsn.WY.2D* (Cui et al., 2012). *QFsn.cib-5A* mapped between 455.42 and 459.97 Mb on chromosome arm 5AL was close to *QFsn.WY.5A.2* detected by Cui et al. (2012).

### Effects on Yield-Related Traits and Potential Use of *QTsn/Fsn.cib-3D*

Similar to previous studies (Chen et al., 2020; Cui et al., 2012), TSN is significantly and positively correlated with FSN. Thus, QTLs, especially the major QTLs, for TSN and FSN were usually identified in same locus. For instance, seven stable QTLs for spikelet number were identified by Chen et al. (2020), and five of them were associated with TSN and FSN simultaneously. The loci of *QTsn/Fsn.czm-1A* and *QTsn/Fsn.czm-1D* were identified by Zhou et al. (2017), showing significant effects on TSN and FSN. A QTL cluster for FSN and TSN identified on chromosome 5D was located in the same region as that detected by Li et al. (2007) and Cui et al. (2012). Moreover, as one of the subcomponent of GNS, an increase of spikelet number could directly increase GNS (Hai et al., 2008). Therefore, it is not surprising that the major QTL *QTsn/Fsn.cib-3D* identified in present study showed significant and positive effects on TSN, FSN, and GNS (Figures 4, 5). Additionally, we further analyzed the effects of *QTsn/Fsn.cib-3D* on other yield-related traits including TKW, SL, PH, GW, and GL in the K1/CM42 populations. *QTsn/Fsn.cib-3D* showed moderate negative influence on GL, but no influence on TKW, PH, SL, and GW (Figure 4). These results suggested that *QTsn/Fsn.cib-3D* combined with developed KASP markers has potential application values in wheat breeding.

### Potential Candidate Genes for *QTsn/Fsn.cib-3D*

In the interval of *QTsn/Fsn.cib-3D*, there are 76 confidence genes in CS (Supplementary Table 3). Based on the expression analysis and gene annotation, five genes *TraesCS3D02G439000*, *TraesCS3D02G439200*, *TraesCS3D02G442000*, *TraesCS3D02G443900*, and *TraesCS3D02G44540*0 are abundantly expressed in spikes, indicating they are likely associated with spike growth and development (Figure 6). Of them, *TraesCS3D02G44540*0 encodes a glutathione *S*-transferase, which has been reported to be related to panicle and spikelet numbers, and plays an important role during the growth and development processes in rice (Kim et al., 2013; Hu et al., 2014). *TraesCS3D02G442000* encodes an auxin response factor. Previous studies have revealed that auxin signal transduction acts critical roles in modulating various biological processes including growth, development, and stress resistance (Qiao et al., 2018; Brauer et al., 2019). *TraesCS3D02G439200* is an ortholog of *OsHAK5* of the rice and encodes a potassium transporter (Supplementary Figure 9). *OsHAK5* plays a crucial role in maintaining rice architecture including PH, root, and tiller by altering cellular chemiosmotic gradients and regulating ATP-dependent polar auxin transport (Yang et al., 2020). *TraesCS3D02G439000* encodes an F-box family protein, which is known to be involved in the vegetative and reproductive growth and development of many plants (Van Den Burg et al., 2008; Ma et al., 2019). *TraesCS3D02G443900* encodes a BTB domain-containing protein, which was reported to be involved in multiple functions including floral organ development and disease resistance in Arabidopsis, rice, maize, and tomato (Gingerich et al., 2005; Chen et al., 2014; Chern et al., 2014; Xu et al., 2016). To further explore the candidate gene, we isolated and assembled the coding sequences of the five genes from four parents. Sequence alignment showed that two non-synonymous SNPs and one synonymous SNP in coding sequence of *TraesCS3D02G443900* were detected between K1 and CM42, and K4 and YFM2; two synonymous SNPs in the coding sequence of *TraesCS3D02G44540*0 were found between K1 and CM42, and K4 and YFM2; and the extra six synonymous SNPs and three non-synonymous SNPs were found between K1 and YFM2, respectively (Figure 7). For the rest three genes *TraesCS3D02G439000*, *TraesCS3D02G439200*, and *TraesCS3D02G442000*, no sequence variation in coding sequences was simultaneously detected between K1 and the other three parents (Supplementary Figures 4, 5, 6). Thus, *TraesCS3D02G443900* and *TraesCS3D02G44540*0 could be considered as a focus for further work on fine mapping and gene cloning.

## Data Availability Statement

The original contributions presented in the study are included in the article/Supplementary Material, further inquiries can be directed to the corresponding author/s.

## Author Contributions

TL undertook the field trials and subsequent analyses of all available data, including the phenotyping and population genotyping, and drafted this manuscript. GD assisted in the field trials. YT, YS, JW, JC, ZY, XQ, XP, HZ, and JL participated in phenotype measurement. MY discussed results and revised the manuscript. HL and YW designed the experiments, guided the entire study, participated in the data analysis, discussed the results, and revised the manuscript. All authors read and reviewed the manuscript.

## Conflict of Interest

The authors declare that the research was conducted in the absence of any commercial or financial relationships that could be construed as a potential conflict of interest.

## Footnotes

1 http://202.194.139.32/

2 https://urgi.versailles.inra.fr/download/

3 https://wheat-urgi.versailles.inra.fr/

4 http://www.uniprot.org/

5 http://wheat.cau.edu.cn/TGT/

6 http://www.wheat-expression.com/

7 http://coultona.cyverseuk.org/
