# Supplementary material for: Identification and Validation of a Novel Locus Controlling Spikelet Number in Bread Wheat (Triticum aestivum L.)
Source: Front Plant Sci. 2021 Feb 26;12:611106. doi: 10.3389/fpls.2021.611106 (PMC7952655; doi:10.3389/fpls.2021.611106)
Supplement: Supplementary file 2 [file Table_2.DOC]

Supplementary Material

# Supplementary Figures


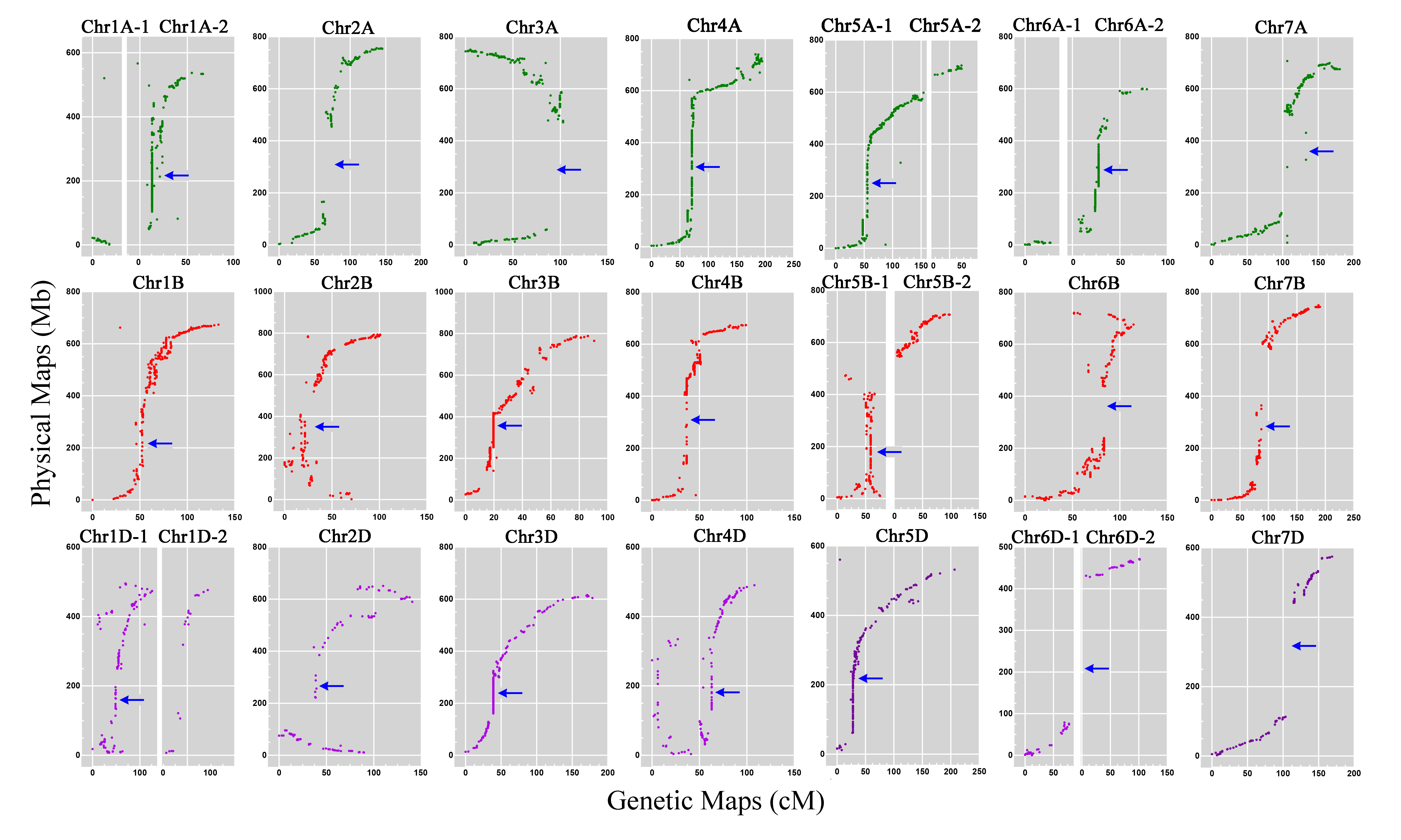


**FIGURE S1.** Comparison of mapped marker orders on the genetic and physical maps. The ordinate represents the physical position of mapped markers, and the abscissa represents the genetic position of mapped markers. Blue arrows indicate the location of the centromere.


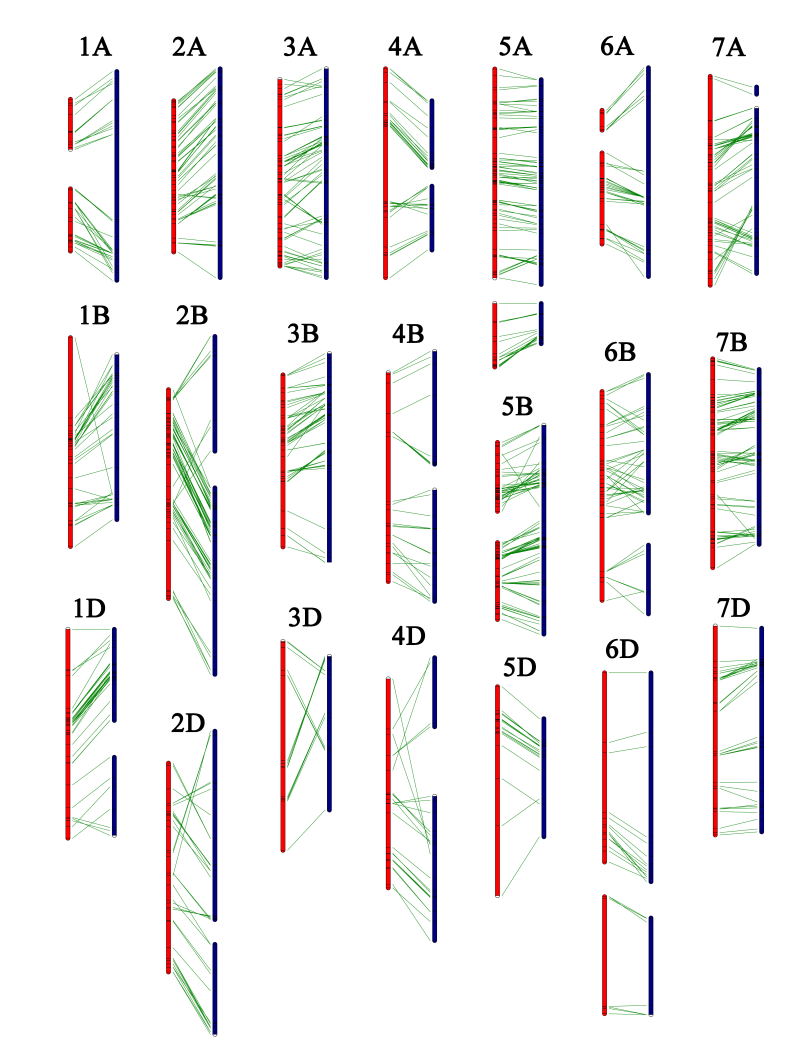


**FIGURE S2.** Correlation of common markers on two genetic maps constructed using the wheat 55 K SNP array. Red bar represents the genetic map constructed in present study; Blue bar represents the genetic map constructed by Liu et al. (2018).


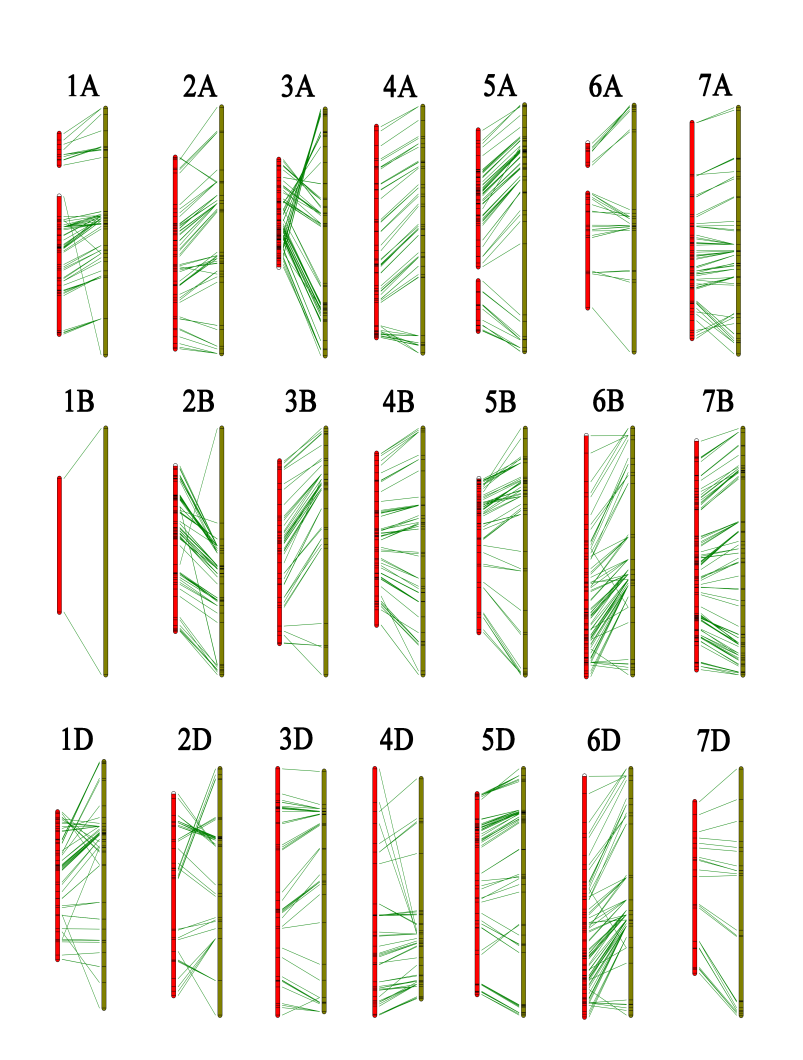


**FIGURE S3.** Correlation of common markers on two genetic maps constructed using the Wheat 55K SNP array and 660K SNP array. Red bar represents the genetic map constructed in present study; Dark olive green bar represents the genetic map constructed by Cui et al. (2017).

**
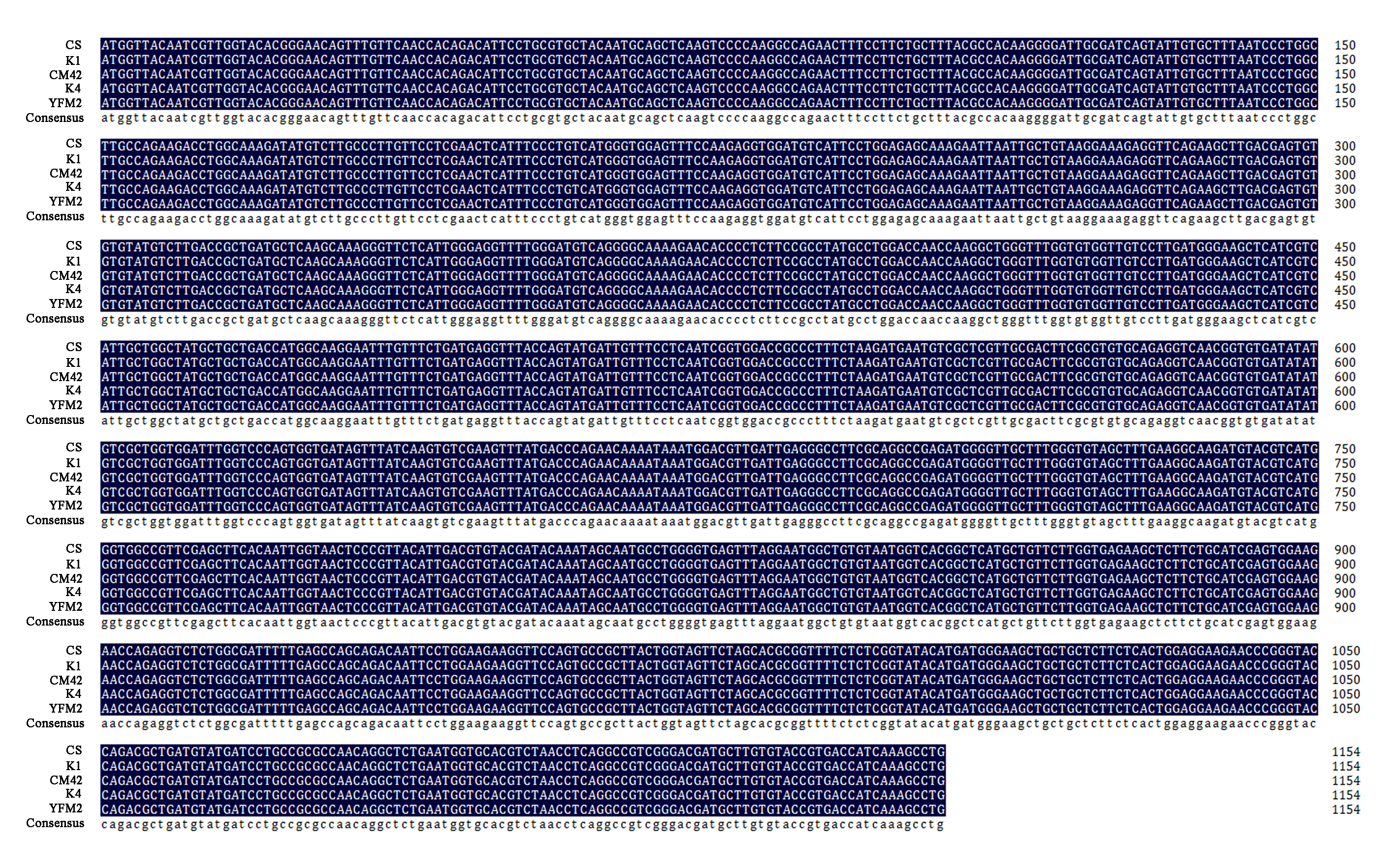
**

**FIGURE S4.** The gene sequence of *TraesCS3D02G439000* from K1, CM42, K4 and YFM2.

**
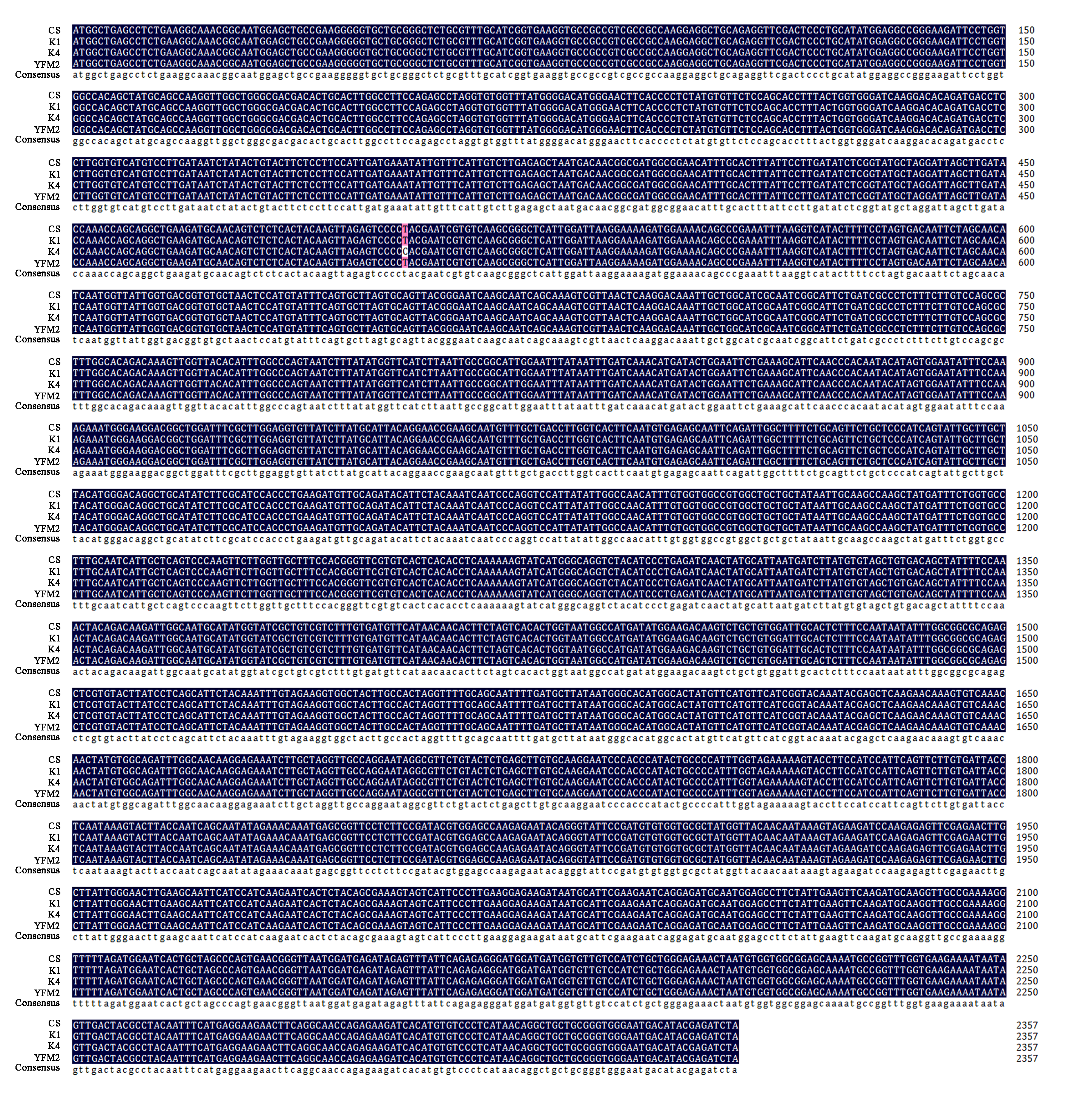
**

**FIGURE S5.** The gene sequence of *TraesCS3D02G439200* from K1, K4 and YFM2.

**
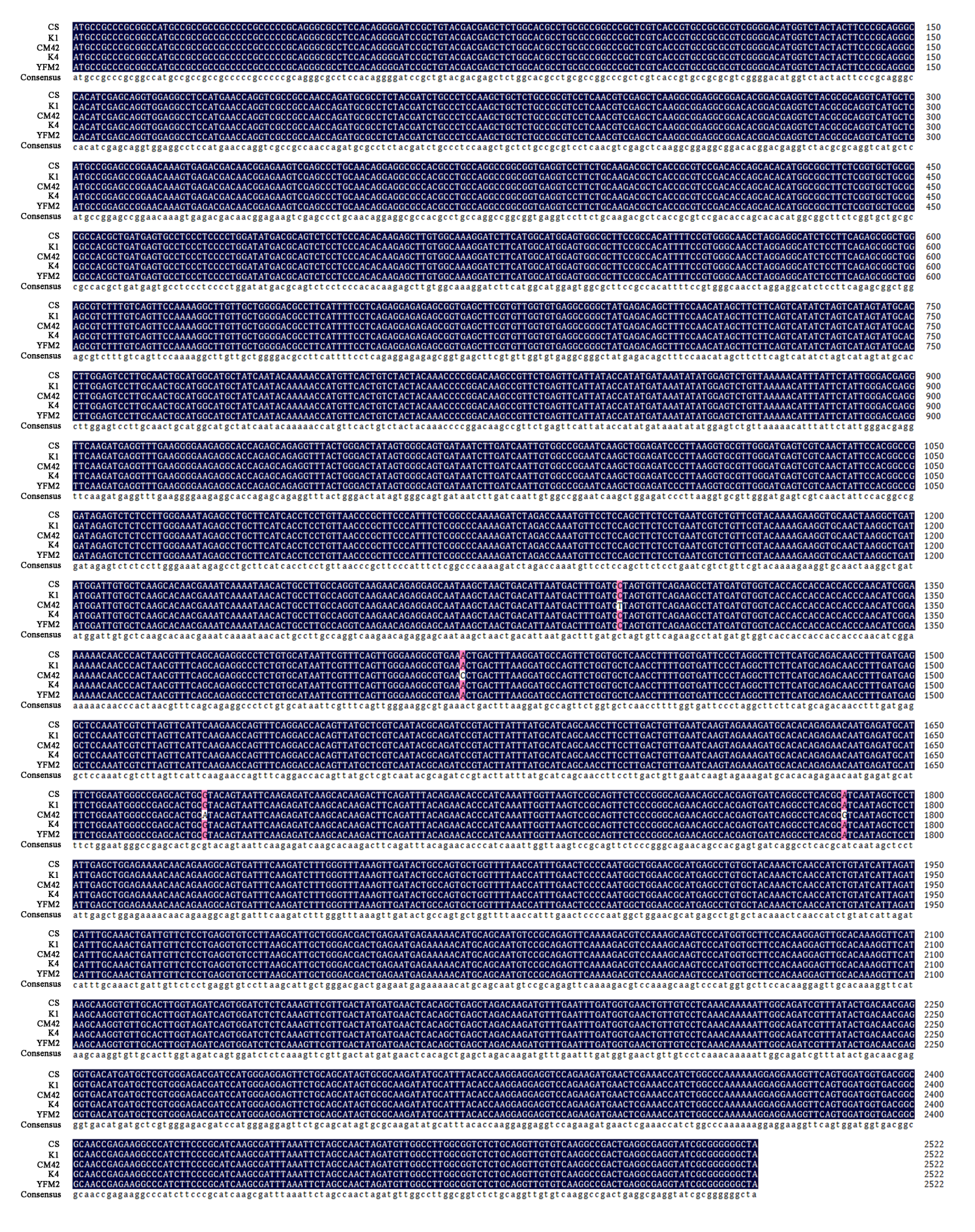
**

**FIGURE S6.** The gene sequence of *TraesCS3D02G442000* from K1, CM42, K4 and YFM2.

**
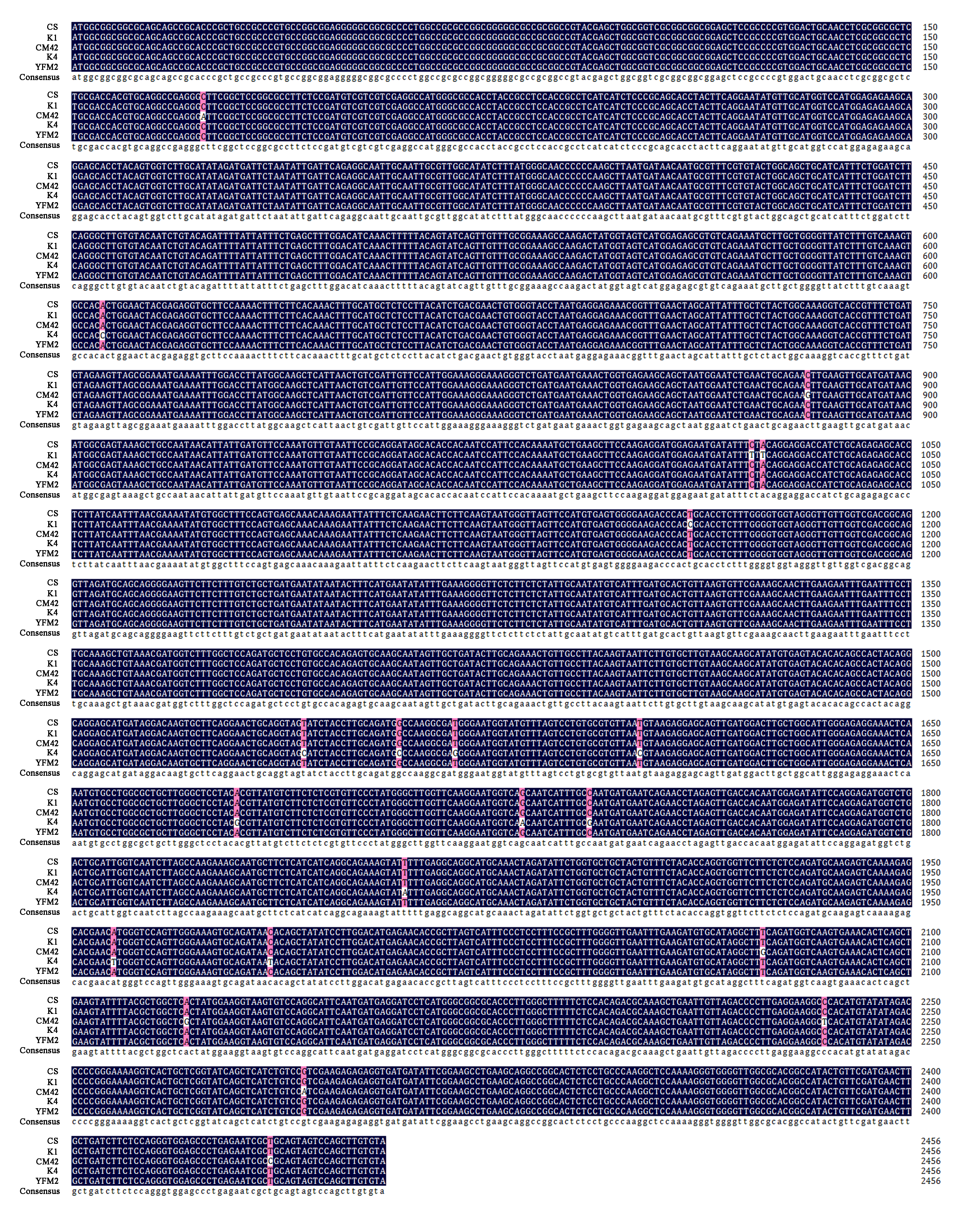
**

**FIGURE S7.** The gene sequence of *TraesCS3D02G443900* from K1, CM42, K4 and YFM2.

**
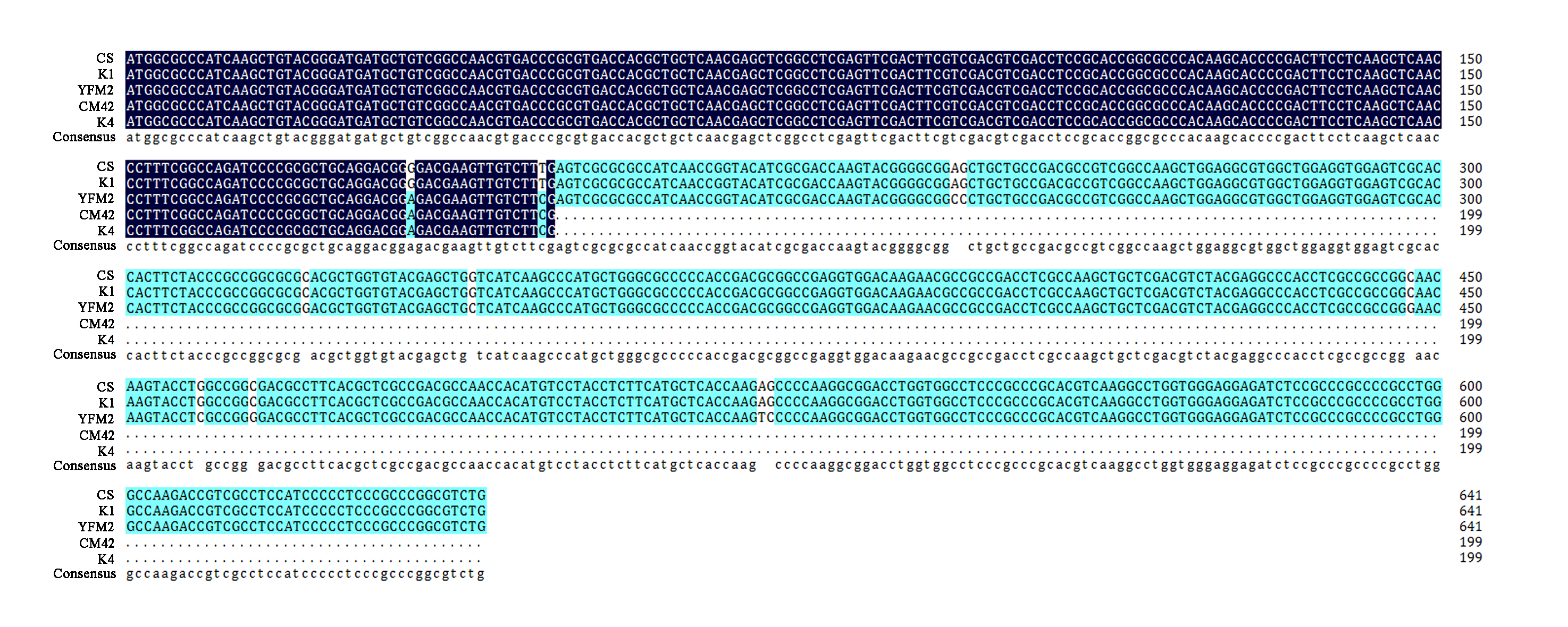
**

**FIGURE S8.** The gene sequence of *TraesCS3D02G445400* from K1, CM42, K4 and YFM2.


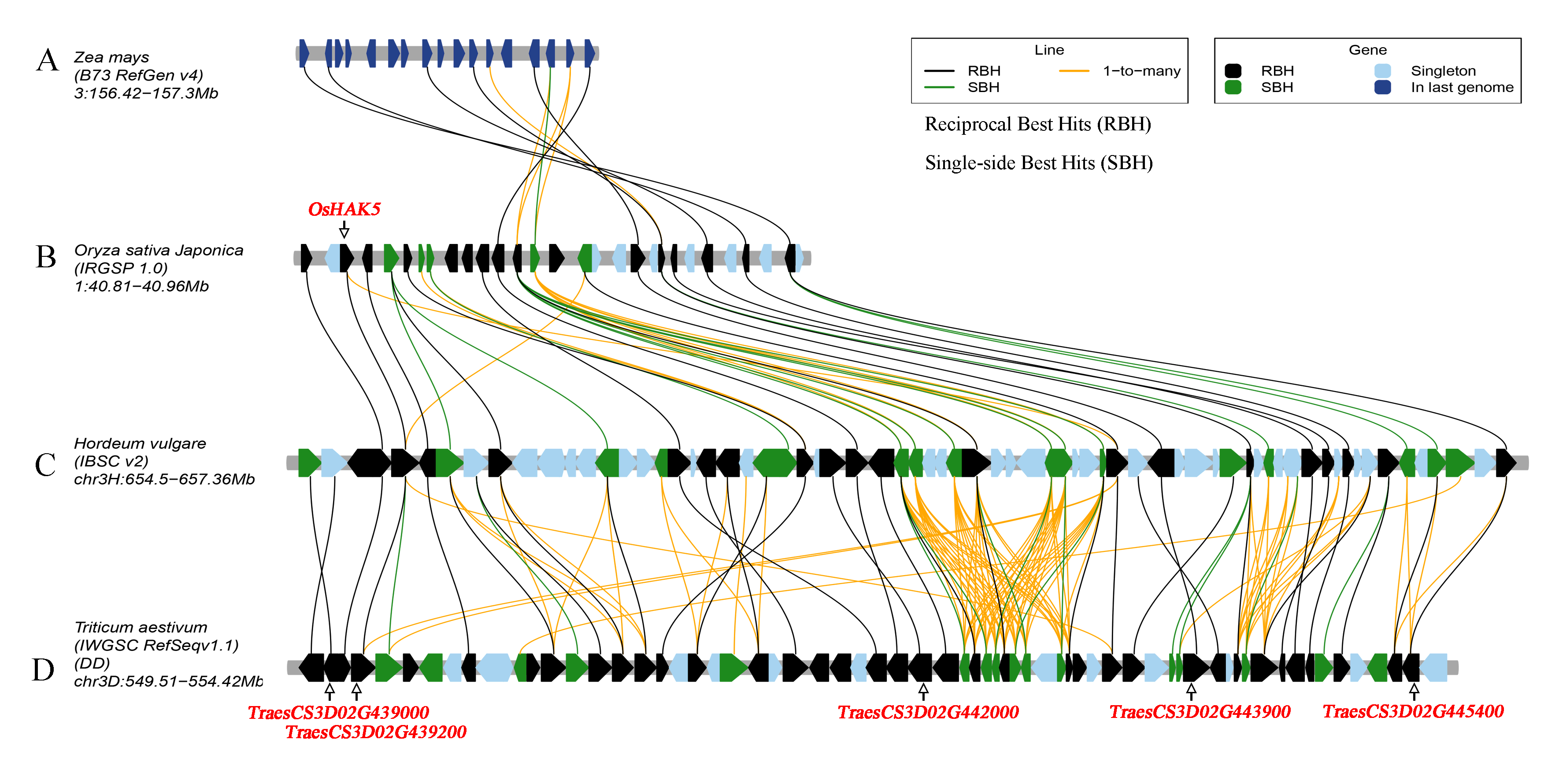


**FIGURE S9.** Gene collinearity among wheat, barley, rice and maize in the physical interval of *QTsn/Fsn.cib-3D*. The collinear regions of 549.51-554.42 Mb on chromosome 3D in wheat (**D**) are 654.5-657.36 Mb on chromosome 3H in barley (**C**), 40.81-40.96 Mb on chromosome 1 in rice (**B**), and 156.42-157.3 Mb on chromosome 3 in maize (**A**); Arrows pointed to the five candidate genes of *QTsn/Fsn.cib-3D* (**D**) and a gene *OsHAK5* that has been cloned in rice (**B**).

**References**

Cui F, Zhang N, Fan X, Zhang W, Zhao CH, Yang LJ, Pan RQ, Chen M, Han J, Zhao XQ, Ji J, Tong YP, Zhang HX, Jia JZ, Zhao GY, Li JM (2017) Utilization of a Wheat660K SNP array-derived high-density genetic map for high-resolution mapping of a major QTL for kernel number. Scientific Reports 7. https://www.nature.com/articles/s41598-017-04028-6

Liu JJ, Luo W, Qin NN, Ding PY, Zhang H, Yang CC, Mu Y, Tang HP, Liu YX, Li W, Jiang QT, Chen GY, Wei YM, Zheng YL, Liu CJ, Lan XJ, Ma J (2018) A 55 K SNP array-based genetic map and its utilization in QTL mapping for productive tiller number in common wheat. Theoretical and applied genetics 131:2439-2450. https://doi.org/10.1007/s00122-018-3164-9
